# Do Birth Outcomes Predicted by Occipital Position Inform Definitions of Occiput Posterior and Occiput Transverse?

**DOI:** 10.7759/cureus.61358

**Published:** 2024-05-30

**Authors:** Angela J Pardey, Hala Phipps, Amanda Eames, Jon Hyett, Sabrina Kuah, Bradley De Vries

**Affiliations:** 1 Department of Obstetrics and Gynaecology, Royal Prince Alfred Hospital, Sydney, AUS; 2 Department of Obstetrics and Gynaecology, Sydney Institute for Women, Children and Their Families, Sydney Local Health District, Sydney, AUS; 3 Department of Obstetrics and Gynaecology, Tweed Valley Hospital, Northern New South Wales (NSW) Local Health District, Cudgen, AUS; 4 Department of Obstetrics and Gynaecology, Western Sydney University, Sydney, AUS; 5 Department of Obstetrics and Gynaecology, The Women's and Children's Hospital, Adelaide, AUS; 6 Faculty of Medicine and Health, The University of Sydney Central Clinical School, Sydney, AUS

**Keywords:** fetal head position, fetal and obstetric outcomes, labour and delivery, human birth, spontaneous vaginal birth, ventouse-assisted birth, forceps-assisted birth, adverse birth outcomes, vaginal birth, birth injuries

## Abstract

Fetal head position significantly influences birth outcomes, with higher rates of complications observed when the fetal head is in the Occiput Posterior (OP) position compared to Occiput Transverse (OT) or Occiput Anterior (OA) positions. There is no consensus in the current literature on the precise rotational point at which the fetal occiput shifts from posterior to transverse, reducing clarity in both scientific and clinical communication. Different studies employ varying definitions of these positions, which affects management decisions.

This study aims to determine if a definable threshold exists between the directly posterior and directly transverse positions that correlates with different birth outcomes, thereby proposing a consistent and clinically useful definition for OP versus OT.

We analyzed ultrasound data from 570 patients at full dilatation from five previous studies, correlating the angle of the fetal occiput (noted on a clock-face) with birth outcomes. Adverse outcomes were defined as cesarean delivery, instrumental vaginal delivery, significant postpartum hemorrhage (500 ml or more), obstetric anal sphincter injury, five-minute Apgar scores <7, arterial cord pH <7, base excess less than -12, or neonatal intensive care unit admission. The analysis was conducted using SAS version 9.4.

The study found a continuous relationship between the fetal occipital angle and adverse birth outcomes without a distinct threshold separating OP from OT positions.

No clear inflection point was demonstrated in pregnancy outcomes between OT and OP. The relationship between the angle of occiput position and pregnancy outcomes was continuous: the closer the fetal head was to directly OP, the higher the likelihood of adverse outcomes. Given the lack of a clear cut-off and to improve consistency in future research, we recommend dividing the occiput position into four quadrants of 90 degrees each. This classification could standardize reporting and potentially improve clinical decision-making regarding fetal position during labor.

## Introduction and background

The occiput posterior (OP) position is present in the second stage of 10% to 20% of all labors [1-5] and has been linked to a range of adverse maternal and perinatal outcomes, including cesarean delivery for slow progress in labor, operative vaginal delivery, labor augmentation, chorioamnionitis, obstetric anal sphincter injury, postpartum hemorrhage, birth trauma, and admission to the neonatal intensive care unit (NICU) [2,5-10]. The occiput transverse (OT) position, present in 10 to 20% of all labors [3, 11-13], is associated with adverse outcomes including cesarean delivery, operative vaginal delivery, prolonged second stage of labor, obstetric anal sphincter injury, febrile morbidity, postpartum hemorrhage, and a low five-minute Apgar score [2,3].

In medical publications, the OP position refers to a fetus with a cephalic presentation whose posterior fontanelle is oriented towards the posterior aspect of the pregnant woman. A direct OP position occurs when the sagittal suture is in the maternal midline. Deviations to the maternal left or right are referred to as left or right OP positions, respectively. However, there is variation in the degree of rotational deviation from the direct OP position that is still considered OP, with definitions varying [1,3,14-19]. Occiput posterior, OT, and OA positions are commonly undefined in the medical literature [2,7,11,20,21].

We considered that the most clinically useful definition of OP position would correlate with the observed perinatal and maternal adverse effects of this position. Therefore, the aims of our study were first to describe the association between the position of the fetal occiput (measured as an angle of deviation from direct OP) and adverse obstetric outcomes, and second, to use this association to determine an optimal definition of OT and OP positions.

## Review

Method

This was a retrospective evaluation of data from two previous observational studies [13,15] and three randomized controlled trials [16,22,23]. For these five studies, investigators were asked to complete a form indicating the fetal position on a clock face. The observational studies compared clinical outcomes by occiput position early in the second stage of labor, whereas the randomized trials assessed the efficacy of manual rotation versus no manual rotation in correcting fetal malposition.

**Figure 1:**
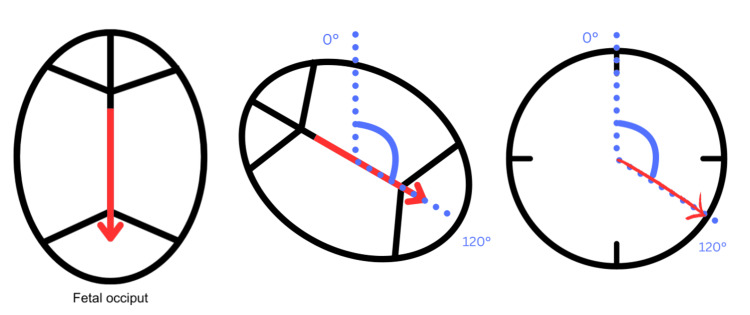
Illustration of the direction of fetal occiput and its angle. A basic illustration showing the top view of the fetal head, highlighting the sutures and fontanelles. The red arrow indicates the specific position on the clock face, showing the degree of deviation from the 12 o'clock (directly vertical) position. The degree of this deviation is the measure documented in our study. This was marked on a 'clock face' diagram similar to the third panel of this figure by investigators in the original studies. The angle illustrated on these forms was subsequently measured to tabulate the data.

**Figure 2:**
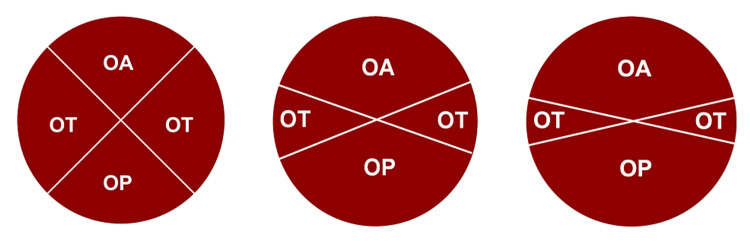
Variations in the definition of occiput posterior position. Left to right: Four quadrants (OT subtends 90°), e.g., Lieberman E et al. (2005) [3], Carseldine WJ et al. (2013) [15], Phipps H. et al. (2021) [16]. OT subtends 45°, e.g., Rane SM et al. (2004) [1], Dupuis O et al. (2005) [14]. OT subtends 30°, e.g., Akmal S et al. (2004) [19], Blasi I et al. (2010) [17], Gizzo S et al. (2014) [18].

Participants randomized to manual rotation were excluded from this study. However, those excluded from the randomized trials because they were in the occiput anterior position were included in this analysis to ensure representation of fetuses in all positions. In total, data from 570 participants were included in our analysis. We aimed to determine which definition of OP versus OT, as illustrated in Figures 1-2, was the most clinically useful in terms of correlation with birth outcomes. Figure 1 demonstrates the directionality of occiput position and how the angles in question were measured.

Population

For the two observational studies [13,15] and one of the randomized trials [22], women gave birth at Royal Prince Alfred Hospital, a tertiary referral hospital in Sydney, Australia, which handles about 5,000 births per year. These studies were conducted between 2008 and 2014. The remaining two randomized trials took place at the same institution and three other sites in Australia from 2012 to 2018 [13,22].

Women were included in the studies if they were at least 37 weeks of gestation with a cephalic singleton pregnancy, planned a vaginal delivery, were at full dilatation, and had the occiput position of the fetal head confirmed by mobile transabdominal ultrasound. Participants were required to be over 16 years old and to have given written informed consent. Women with any fetal position were included in the observational studies [13,15], and women with OP [16] or OT positions [23] were included in the randomized trials. However, the current study included women who provided informed consent for the randomized trials but were later excluded due to an occiput anterior (OA) position. Exclusion criteria varied slightly between studies but generally included factors considered to increase the risk of emergency cesarean delivery such as antepartum hemorrhage, abnormal cardiotocography, or evidence of fetal acidemia [13,15,16,22,23]. Women with a previous cesarean delivery were excluded from all studies. For the randomized trials, women allocated to the treatment group - manual rotation in labor - were also excluded from the current study as manual rotation could have affected maternal and perinatal outcomes.

For descriptive purposes, four quadrants of fetal occiput position were defined as follows: Left occiput anterior/left occiput transverse (LOA/LOT) from 0° to <90°; Right occiput anterior/right occiput transverse (ROA/ROT) from ≥90° to <180°; Left occiput posterior/left occiput transverse (LOP/LOT) from ≥180° to <270°; Right occiput posterior/right occiput transverse (ROP/ROT) from ≥270° to <360°; and direct positions were also defined for OA (0°), LOT (90°), OP (180°), and ROT (270°).

Outcomes

The impact of occipital position was assessed for several obstetric outcomes including delivery by cesarean section, operative (vacuum or forceps) delivery, or the need for intervention to effect delivery (either caesarean section or operative vaginal delivery). Additional outcome measures were obstetric anal sphincter injury (OASI) - defined clinically as a third- or fourth-degree tear, postpartum hemorrhage (defined as blood loss >500 ml associated with delivery), a five-minute Apgar score <7, an abnormal arterial cord gas result (pH <7.1 or base excess less than -12 mEq/L), and admission to the neonatal intensive care unit. The combined adverse maternal outcome included operative delivery, postpartum hemorrhage, or third/fourth degree perineal trauma. The combined adverse perinatal outcome included abnormal cord blood gases, a five-minute Apgar score <7, or admission to the neonatal intensive care unit. Finally, all factors were considered as a composite adverse outcome.

Statistical analysis

Data were analyzed using SAS version 9.4 (SAS Institute, Inc., Cary, NC, USA). Categorical data were expressed as percentages, and continuous data as means and SDs if normally distributed, or as medians and interquartile ranges otherwise.

Multivariable logistic regression was conducted for the composite adverse outcome measure as well as for perinatal and maternal combined adverse outcomes. Stepwise backward elimination was used, with variables removed if p > 0.05 and no significant impact was observed on the odds ratios for fetal occiput position. Multiple imputation was not used. A post-hoc decision was made to repeat the regression analysis for operative delivery as the outcome, as it was considered that this might drive any associations with our combined outcome measures.

Approval was obtained from the ethics committee at Royal Prince Alfred Hospital, Sydney, Australia, for all studies contributing data to the current analysis (protocol numbers X07-0173 [13,15], X10-0091 [13,22], and X11-0410 [16,23]).

Results

**Figure 3:**
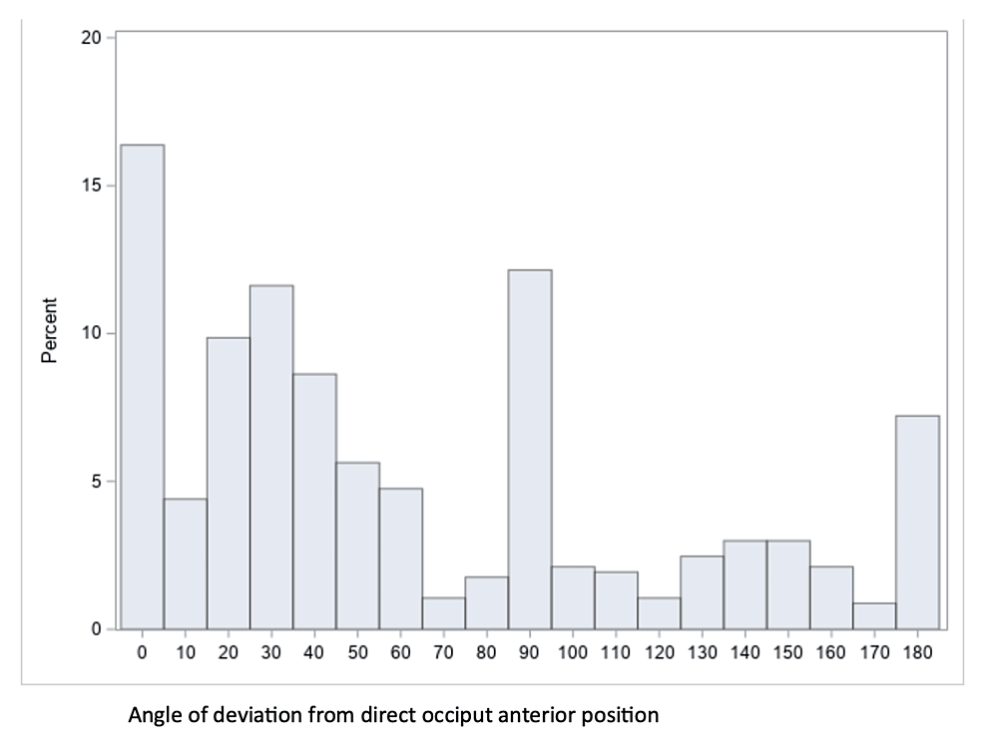
Histogram showing degrees of deviation from the direct occiput anterior position.

Fetal position with the angle of rotation from direct OP position was available for 570 participants. For the four quadrants, the distribution was 26% (147 participants) for the LOA/LOT position, 26% (148 participants) for the ROA/ROT position, 8.6% (49 participants) for the LOP/LOT position, and 10% (59 participants) for the ROP/ROT position. Thirteen percent (73 participants) were in direct OA, 6.3% (36 participants) were in direct OP, 4.2% (24 participants) were in direct LOT, and 5.6% (32 participants) were in direct ROT. Figure 3 shows a histogram of the degrees of deviation from the direct OA position, with zero degrees representing direct OA and 180 degrees representing direct OP positions.

Table 1 displays baseline characteristics by subtended angles of deviation, divided into four groups of 45 degrees. Baseline characteristics were similar across all four groups.

**Table 1:** Baseline characteristics of 570 women with no previous caesarean section with ultrasound-determined occiput posterior position in the second stage of labour.

	0-45°	>45-90°	>90-135°	>135-180°
n	289	81	108	92
Nulliparous (n %)	234 (81%)	66 (84%)	93 (86%)	72 (78%)
Age (median, IQR)	31 (5.8)	31 (7.09)	30 (6.72)	30 (6.78)
BMI (median, IQR)	23.39 (6.33)	23.14 (4.90)	24.30 (7.51)	24.00 (6.16)
Missing data for BMI	62 (21.45%)	17 (20.98%)	17 (15.74%)	11 (11.95%)
Neonatal sex female (n %)	150 (51.9%)	35 (43.21%)	54 (50%)	50 (54.35%)
Birthweight (mean, SD)	3445.47g (419.56)	3500.32g (444.56)	3507.94g (474.74)	3457.51g (407.50)

**Figure 4:**
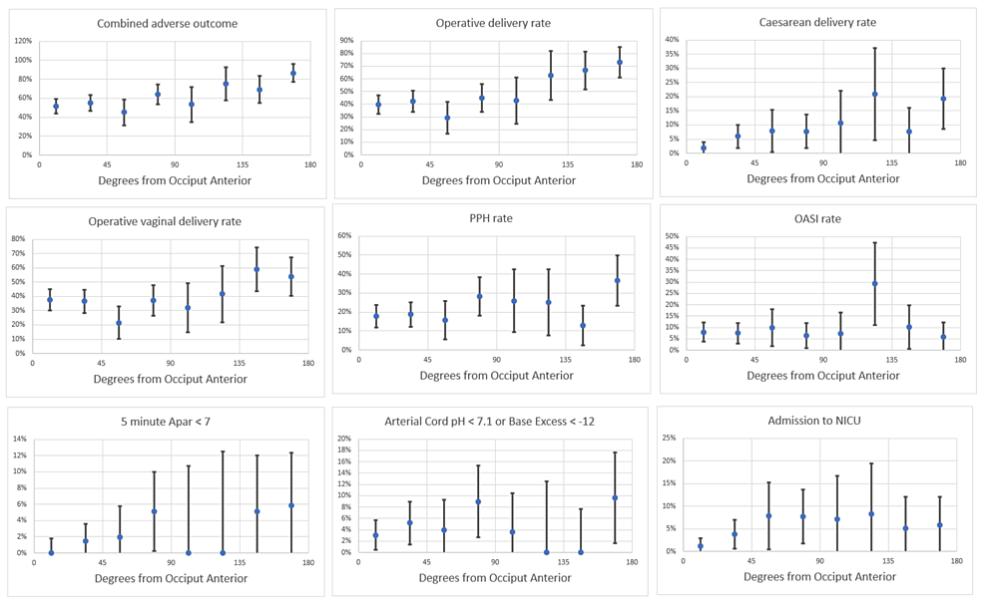
Adverse outcomes by degrees of deviation from the direct occiput anterior position among 570 women with no previous caesarean section in the second stage of labour: all women. Graphs showing point estimates and 95% confidence intervals. Plotted angles subtend 22.5°: 0 to <22.5°, 22.5° to <45°, 45° to <67.5°, 67.5° to <90°, 90° to <112.5°, 112.5° to <135°, 135° to <157.5°, and 157.5° to 180°.

**Figure 5:**
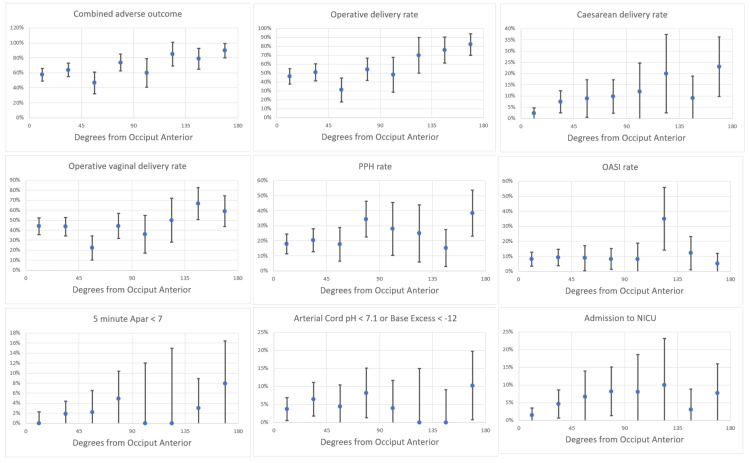
Adverse outcomes by deviation from the direct occiput anterior position among 464 women with no previous cesarean in the second stage of labor: nulliparous women only. Graphs show point estimates and 95% confidence intervals. Plotted angles subtend 22.5°: 0 to <22.5°; 22.5° to <45°; 45° to <67.5°; 67.5° to <90°; 90° to <112.5°; 112.5° to <135°; 135° to <157.5°; and 157.5° to <180°.

Figures 4-5 illustrate point estimates and 95% confidence intervals (CIs) for combined adverse maternal and perinatal outcomes by angle of deviation from direct OA for all women in the cohort (Figure 4) and for nulliparous women only (Figure 5). Adverse outcomes gradually increased with increasing deviation from direct OA, with no clear inflection point that could be used to define OP or OT positions.

Table 2 shows the raw numbers of adverse outcomes and point estimates for percentages that correspond to the graphical representation in Figures 3-4.

**Table 2:** Maternal and perinatal outcomes among 570 women with no previous cesarean section in the second stage of labor. ^Defined as one or more of: cesarean delivery, instrumental vaginal delivery (vacuum or forceps), postpartum hemorrhage ≥ 500 mL, obstetric anal sphincter injury (3rd or 4th degree perineal injury), five-minute Apgar score < 7, arterial cord pH < 7.1 or base excess < -12 mEq/L, admission to the neonatal intensive care unit. *pH < 7.1 or BE <-12 mEq/L Note: These data correspond with the graphical representation in Figure 5. OVD: Operative vaginal delivery; OASI: Obstetric Anal Sphincter Injury (third or fourth degree perineal tears); PPH: Postpartum Haemorrhage (defined here as a blood loss of 500mL or more);  NICU: Neonatal Intensive Care Unit.

	0-22.5°	>22.5-45°	>45-67.5°	>67.5-90°	>90-112.5°	>112.5-135°	>135-157.5°	>157.5-180°
Nulliparous women (n = 465) n (%)								
n	134	108	45	61	25	20	33	39
Combined adverse outcome^	77 (57)	69 (64)	21 (47)	45 (74)	15 (60)	17 (85)	26 (79)	35 (90)
Caesarean delivery	2 (2.2)	8 (7.4)	4 (8.9)	6 (9.8)	3 (12)	4 (20)	3 (9.1)	9 (23)
OVD*	59 (44)	47 (44)	10 (44)	27 (44)	9 (36)	10 (50)	22 (66.7)	23 (59)
OASI	11 (8.2)	10 (9.3)	4 (8.9)	5 (8.2)	2 (8)	7 (35)	4 (12)	2 (5.1)
PPH >500mL	24 (18)	22 (20)	8 (18)	21 (34)	7 (28)	5 (25)	5 (15)	15 (38)
Apgar score <7	0 (0)	2 (1.8)	1 (2.2)	3 (4.9)	0 (0)	0 (0)	1 (3)	3 (7.9)
NICU Admission	2 (1.5)	5 (4.6)	3(6.7)	5 (8.2)	2 (8)	2 (10)	1 (3)	3 (7.7)
Abnormal arterial cord blood gases**	5 (3.7)	7 (6.5)	2 (4.4)	5 (8.2)	1 (4)	0 (0)	0 (0)	4 (10)
Parous women (n = 105) n (%)								
n	30	26	6	17	3	4	6	13
Combined adverse outcome^	8 (27)	5 (19)	2 (33)	5 (29)	0 (0)	1 (25)	1 (17)	10 (77)
Caesarean delivery	0 (0)	0 (0)	0 (0)	0 (0)	0 (0)	1 (25)	0 (0)	1 (7.7)
OVD	3 (10)	2 (7.7)	1 (17)	2 (12)	0 (0)	0 (0)	1 (16)	5 (38)
OASI	2 (6.7)	0 (0)	1 (16.7)	0 (0)	0 (0)	0 (0)	0 (0)	1 (7.7)
PPH >500mL	5 (17)	3 (11)	0 (0)	1 (5.9)	0 (0)	1 (25)	0 (0)	4 (31)
Apgar score <7	0 (0)	0 (0)	0 (0)	1 (5.9)	0 (0)	0 (0)	1 (17)	0 (0)
NICU Admission	0 (0)	0 (0)	1 (17)	1 (5.9)	0 (0)	0 (0)	1 (17)	0 (0)
Abnormal arterial cord blood gases**	0 (0)	0 (0)	0 (0)	2 (12)	0 (0)	0 (0)	0 (0)	1 (7.7)

Table 3 shows the results of the multivariable logistic regressions. The continuous variables (fetal occiput position, maternal age, BMI, and birthweight) were categorized into four groups due to non-linearity. Deviation from the direct OA position was associated with combined adverse maternal/perinatal outcomes, combined maternal adverse outcomes, and operative delivery after adjusting for confounders.

**Table 3:** Logistic regression for combined adverse maternal and perinatal outcomes among 570 women with no previous cesarean section in the second stage of labor. * All variables in the following list were excluded in the backward elimination due to p > 0.05 and not affecting the point estimates of odds ratios for angle of occiput from direct OA (except for those shown in the table): maternal age group, birthweight group, body mass index group, side of fetal occiput (maternal left, right or midline), induced v spontaneous labour, neonatal sex, and parity. ^Referent group aOR: adjusted odds ratio.

Variable	aOR (95%CI)	P-value
Combined adverse outcome*		
Angle of occiput from direct OA		<0.0001
0 to <45°	1.0^	
45 to < 90°	0.80 (0.47-1.3)	
90 to < 135°	1.5 (0.94-2.4)	
135 to < 180°	3.8 (2.3-6.8)	
Parity		<0.0001
Nulliparous	1.0^	
Parous	0.21 (0.13-0.34)	
Neonatal sex		0.046
Female	1.0^	
Male	1.4 (1.007-2.1)	
Perinatal adverse outcome*		
Angle of occiput from direct OA		0.073
0 to <45°	1.0^	
45 to < 90°	3.1 (1.2-8.5)	
90 to < 135°	2.8 (1.1-7.4)	
135 to < 180°	2.7 (0.96-7.5)	
Neonatal sex		0.0014
Female	1.0^	
Male	4.0 (1.7 – 9.5)	
Maternal adverse outcome*		
Angle of occiput from direct OA		< 0.0001
0 to <45°	1.0^	
45 to < 90°	0.70 (0.41-1.2)	
90 to < 135°	1.3 (0.84-2.1)	
135 to < 180°	3.8 (2.2-6.9)	
Parity		<0.0001
Nulliparous	1.0^	
Parous	0.19 (0.11-0.31)	
Operative delivery*		
Angle of occiput from direct OA		< 0.0001
0 to <45°	1.0^	
45 to < 90°	0.69 (0.40-1.2)	
90 to < 135°	1.3 (0.82-2.1)	
135 to < 180°	4.2 (2.4-7.2)	
Parity		<0.0001
Nulliparous	1.0^	
Parous	0.13 (0.07-0.24)	

Our data demonstrated that adverse maternal and perinatal outcomes increased with increasing deviation from direct OA, with no clear cutoff for defining OP or OT positions early in the second stage of labor. The results are consistent with observational data showing an increase in adverse outcomes for OT and OP positions compared to the OA position [13,15]. These findings are significant because there is inconsistency in definitions for OP and OT positions in the medical literature [1,3,14-19], and many publications do not define the OP position at all [2,7,11,20,21]. The occiput posterior position has been variously defined to include an arc encompassing anywhere from 45 degrees to 75 degrees from the midline, and the OT position anywhere from 15 to 45 degrees from the direct transverse position (Figure 2).

Discussion

Our main finding was that adverse maternal and perinatal outcomes increased with increasing deviation of the occiput position from direct OA, without a clear cutoff for defining OP or OA positions early in the second stage of labor. This is significant due to the well-demonstrated impact of fetal head position on birth outcomes and the need for clear, consistent definitions in both the literature and clinical communication at the point of care.

Currently, no published studies have specifically examined the impact of fetal occiput position angles on maternal and perinatal outcomes, making it challenging to compare and discuss our findings.

While this study does not provide a clear cutoff for definitions of OA, OT, and OP positions, we suggest that the simplest definition involves dividing the fetal occiput position into four equal quadrants, as shown in the left-most depiction in Figure 2. Future consistency in definitions will facilitate comparison of results across studies of fetal malposition, including the impact on clinical outcomes such as operative birth, for diagnostic accuracy studies such as assessing the accuracy of vaginal examination for diagnosing OP position, and for treatment studies such as assessing the rate of ‘successful’ manual rotation from the OP or OT position to the OA position.

We also found that the occiput position was strongly associated with adverse combined and maternal outcomes after adjusting for confounders, as shown in Table 3. This finding was primarily driven by an increase in operative deliveries. Not surprisingly, male neonatal sex was associated with adverse perinatal outcomes, and nulliparity was associated with adverse maternal outcomes, primarily due to higher rates of operative delivery in nulliparous women. However, even after adjusting for parity, the occiput posterior position was strongly associated with adverse maternal outcomes, especially operative delivery, and can be considered an independent predictor.

The strengths of this study include the use of ultrasound to assess fetal position in the second stage of labor, which is known to be more accurate than vaginal assessment, and the inclusion of participants across multiple (four) centers, increasing the generalizability of our findings. Another strength is that this is the first published study on the precise definition of OP versus OT. Limitations include that our results are limited to the fetal occiput position early in the second stage of labor, meaning that results may not apply to the antenatal or early labor settings or the point of delivery. Another limitation is that women undergoing rapid labors may have proceeded to have a vaginal birth before there was an opportunity to call in a study investigator to perform an ultrasound. This could weaken the observed relationship between occiput position and adverse outcomes, as the more rapid labors not included in the study would be more likely to have a fetus in the OA position. It could also explain the relatively high instrumental delivery rates for OA positions. The precision of the angle data is somewhat limited due to the subjective nature of evaluating fetal position and the variability in managing malposition during labor. Even with ultrasound, investigators may tend to round the position to major cardinal directions, which would explain the peaks in the number of babies whose positions were directly OP and OT compared to those just a few degrees off (see Figure 3). However, the study's large sample size likely mitigates the margin of error, and the study’s inexactness mirrors the degree of inexactness present in clinical practice.

## Conclusions

In conclusion, as the occiput position deviates further from direct OA, adverse obstetric outcomes become increasingly common. A clear cutoff for defining OA, OT, and OP positions could not be established. Therefore, we recommend dividing the occiput position into four equal-sized quadrants of 90 degrees each. This approach will promote consistency in reporting for future studies.
